# Assessment of left atrial function in feline hypertrophic cardiomyopathy by using two- dimensional speckle tracking echocardiography

**DOI:** 10.1186/s12917-020-02557-3

**Published:** 2020-09-18

**Authors:** Arisara Kiatsilapanan, Sirilak Disatian Surachetpong

**Affiliations:** Department of Veterinary Medicine, Faculty of Veterinary Science, Chulalongkorn University, 10330 Bangkok, Thailand

**Keywords:** Cats, Heart, Left atrium, Strain

## Abstract

**Background:**

Left atrial (LA) function plays an important role in diastolic dysfunction in cats with hypertrophic cardiomyopathy (HCM). Two-dimensional speckle tracking echocardiography (2D-STE) is a technique for assessing LA function. This study aimed to evaluate the LA function in HCM cats compared to normal cats, using 2D-STE.

**Results:**

Seventeen client-owned cats affected with HCM and twenty healthy control cats were studied. Conventional echocardiographic and 2D-STE variables were measured and compared between groups (control and HCM groups). Variability of the peak atrial longitudinal strain (PALS) displayed good reproducibility with 4.7% intra-observer and 14% inter-observer repeatability. The mean value of PALS in the HCM group (13.16 ± 8.64) was lower than that in the control group (28.54 ± 10.31) (*P* < 0.001). PALS was lowest in the LA roof region. The atrial longitudinal strains of septal and lateral regions were significantly lower in the HCM group than in the normal group. The PALS correlated with the percentage of the LA fractional shortening (LA-FS) (r = 0.538, *P* = 0.001), the percentage of the LA ejection fraction (LA-EF) (r = 0.797, *P* < 0.001), and the LA fractional area change (FAC) (r = 0.746, *P* < 0.001).

**Conclusions:**

PALS is a feasible and reproducible method to evaluate the LA function in cats affected with HCM.

## Background

Hypertrophic cardiomyopathy (HCM) is one of the most common myocardial diseases. The prevalence of HCM in cats was approximately 10–15% in cats and increased with age [1–4]. The American College of Veterinary Internal Medicine consensus guideline of cardiomyopathies in cats suggests that left ventricular wall thickness during diastole of ≥ 6 is indicative of left ventricular hypertrophy [5, 6]. Hypertrophic cardiomyopathy is associated with diastolic dysfunction [5]. An advanced diastolic dysfunction is related to clinical signs [2]. Some HCM cats may develop congestive heart failure, arterial thromboembolism, and sudden death [3, 7].

The left atrium (LA) plays an important role in cardiac performance through its three phasic functions: reservoir, conduit, and booster pump functions [8–12]. The first is the reservoir phase, in which the LA obtains blood from the pulmonary venous flow during the left ventricular systole. The second is the conduit phase, when the LA transfers blood passively into the left ventricle during early diastole. The final phase is the booster pump, which represents LA contraction during the late diastole [8–12]. Assessment of LA size is the most important indicater of the chronicity, severity and progression of the disease [5].

Several methods have been used in the assessment of LA function in humans and dogs, such as echocardiography, magnetic resonance imaging and computed tomography [13–19]. Echocardiographic techniques used for evaluating LA function in humans, dogs, and cats include phasic volume changes and tissue Doppler imaging, but these techniques have limitations, including load- and angle-dependence, and the tethering effect [5, 11, 14, 20–25].

Two-dimensional speckle tracking echocardiography (2D-STE) is an echocardiographic technique that can be used to assess LA function by tracking acoustic speckle patterns of the LA wall, and to analyse the myocardial motion [10, 11, 26]. Two-dimensional speckle tracking echocardiography is a feasible and reproducible method for evaluating LA wall deformation and can be used together with conventional echocardiography to assess LA function. [10, 11, 26].

Two-dimensional speckle tracking echocardiography has been utilized to assess LA function in humans and dogs [8–12, 26, 27]. Few studies using 2D-STE to assess left ventricular function in HCM cats have been published [22, 23, 28–30]. To our knowledge, there are no studies focusing on the assessment of LA function by 2D-STE in feline HCM. We hypothesized that changes in LA function of HCM cats can be detected using 2D-STE. This study aimed to use 2D-STE to evaluate changes in LA function in HCM cats and compare them to those of healthy control cats.

## Results

A total of 37 cats were included in the study, including 20 control cats and 17 HCM cats. The general characteristics of the control and HCM groups are summarized in Table 1. Age, body weight, heart rate and systolic blood pressure did not differ significantly between the control and HCM groups. Male and domestic shorthair cats were over-represented, but the number of cats in each sex and breed did not differ significantly between the control and HCM groups. Five of 17 cats in the HCM group had systolic anterior motion assessed by echocardiography. None of cats recruited to the study had mitral valve dysplasia which may be the cause of left ventricular outflow tract obstruction and left ventricular hypertrophy.

**Table 1:** The general characteristics of cats in the control and hypertrophic cardiomyopathy groups

Variable	Control (*N* = 20)	HCM (*N* = 17)	*p*-value
Age (year)	5.05 ± 3.03	5 ± 3.43	0.963
Body weight (Kg)	4.47 ± 0.92	4.25 ± 1.04	0.497
Sex Male Female	14 6	10 7	0.512
Heart rate (bpm)	206 ± 20	209 ± 31	0.798
Systolic blood pressure (mmHg)	128 ± 20	116 ± 20	0.073
Breed Domestic Shorthair American shorthair Sphinx Scottish fold Siamese Exotic shorthair Persian Khao Manee	10 5 1 2 1 1 - -	10 - - - - 1 5 1	0.843

Age, body weight, heart rate and systolic blood pressure are expressed as mean ± standard deviation

Sex and breeds are expressed as the number of cats

Numerical data were compared by using the independent student t-test

Categorical data were compared by using Fisher’s Exact test

*HCM* Hypertrophic cardiomyopathy

The result of the conventional echocardiography showed an increase in LAD and the ratio of LA to aorta dimension in the HCM group (*P* < 0.001) compared to the control group, while left ventricular internal dimension at end-diastole were significantly lower in the HCM group than in the control group (*P* = 0.027). The pulsed-wave Doppler echocardiography demonstrated that peak velocity of early diastolic transmitral flow (*P* = 0.002), peak velocity of systolic pulmonary vein flow (*P* = 0.001), and peak velocity of diastolic pulmonary vein flow (*P* = 0.006) were significantly lower in the HCM group than the control group. The pulsed-wave Doppler echocardiography and tissue Doppler imaging were not significantly different between the two groups. The LA-FS (*P* < 0.001), LA-EF (*P* = 0.001) and FAC (*P* < 0.001) were significantly lower in the HCM group than in the control group (Table 2).

**Table 2:** Comparison of conventional echocardiographic values in the control and hypertrophic cardiomyopathy groups

Variable	Control (*N* = 20)	HCM (*N* = 17)	*p*-value
**Size and structure**			
** IVSd(mm)**	4.24 ± 0.89	7.12 ± 1.14	< 0.001*
LVIDd(mm)	14.31 ± 1.65	12.38 ± 3.03	0.027*
LVPWd(mm)	3.5 ± 0.59	5.56 ± 1.64	< 0.001*
IVSs(mm)	7.18 ± 1.40	8.63 ± 1.86	0.01*
LVIDs(mm)	6.35 ± 1.63	6.06 ± 2.38	0.669
LVPWs(mm)	7.01 ± 0.92	8.24 ± 1.94	0.026*
LA (mm)	12.22 ± 1.42	15.55 ± 2.81	< 0.001*
Ao (mm)	8.66 ± 1.3	8.3 ± 1.73	0.476
LA:Ao	1.44 ± 0.28	1.92 ± 0.44	< 0.001*
**LV function**			
FS%	55.58 ± 10.22	51.38 ± 10.79	0.233
E (m/s)	0.83 ± 0.19	0.62 ± 0.18	0.002*
A (m/s)	0.61 ± 0.12	0.53 ± 0.29	0.346
E:A	1.34 ± 0.31	1.46 ± 0.74	0.09
IVRT (m/s)	45.7 ± 6.64	47.4 ± 9.12	0.504
S (cm/s)	0.46 ± 0.09	0.31 ± 0.13	0.001*
D (cm/s)	0.35 ± 0.06	0.26 ± 0.12	0.006*
AR (cm/s)	0.14 ± 0.03	0.16 ± 0.05	0.367
S:D ratio	1.25 ± 0.2	1.2 ± 0.22	0.464
E′ (m/s)	0.11 ± 0.03	0.08 ± 0.04	0.056
A′ (m/s)	0.08 ± 0.03	0.07 ± 0.04	0.54
S′ (m/s)	0.07 ± 0.02	0.07 ± 0.03	0.625
E′:A′ ratio	1.33 ± 0.31	1.15 ± 0.39	0.16
E:E′ ratio	7.8 ± 2.92	9.7 ± 6.8	0.265
**LA function**			
LA-FS (%)	27.27 ± 6.94	14.95 ± 8.23	< 0.001*
LA-EF (%)	69.10 ± 13.64	46.09 ± 21.48	0.001*
FAC (%)	63.73 ± 11.30	35.54 ± 18.07	< 0.001*

*A* peak velocity of early diastolic transmitral flow, *A′* peak velocity of diastolic mitral annular motion as determined by pulsed wave Doppler, *Ao* Aorta, *AR* peak velocity of pulmonary vein flow reversal at atrial contraction, *D* peak velocity of diastolic pulmonary vein flow, *E* peak velocity of early diastolic transmitral flow, *E′* peak velocity of early diastolic mitral annular motion as determined by pulsed wave Doppler, *E:A* ratio of E to A, *E′A′* ratio of E′ to A′, *E:E′* ratio of E to E′, *FAC* left atrial fractional area change, *FS* left ventricular fractional shortening, *HCM* hypertrophic cardiomyopathy, *IVRT* isovolumic (or isovolumetric) relaxation time, *IVSd* interventricular septum thickness at end-diastole, *IVSs* interventricular septum thickness at end-systole, *LA* left atrium, *LA:Ao* left atrial and aorta ratio, *LA-EF* left atrial ejection fraction, *LA-FS* left atrial fractional shortening, *LVIDd* left ventricular internal dimension at end -diastole, *LVIDs* left ventricular internal dimension at end -systole, *LVPWd* left ventricular posterior wall thickness at end-diastole, *LVPWs* left ventricular posterior wall thickness at end-systole, *S* peak velocity of systolic pulmonary vein, *S′* peak velocity of systolic mitral annular motion as determined by pulsed wave Doppler, *S:D* ratio of S to D

Data are expressed as mean ± standard deviation

* indicate statistical significance at *p* < 0.05 assessed by the independent student t-test

**Fig. 1:**
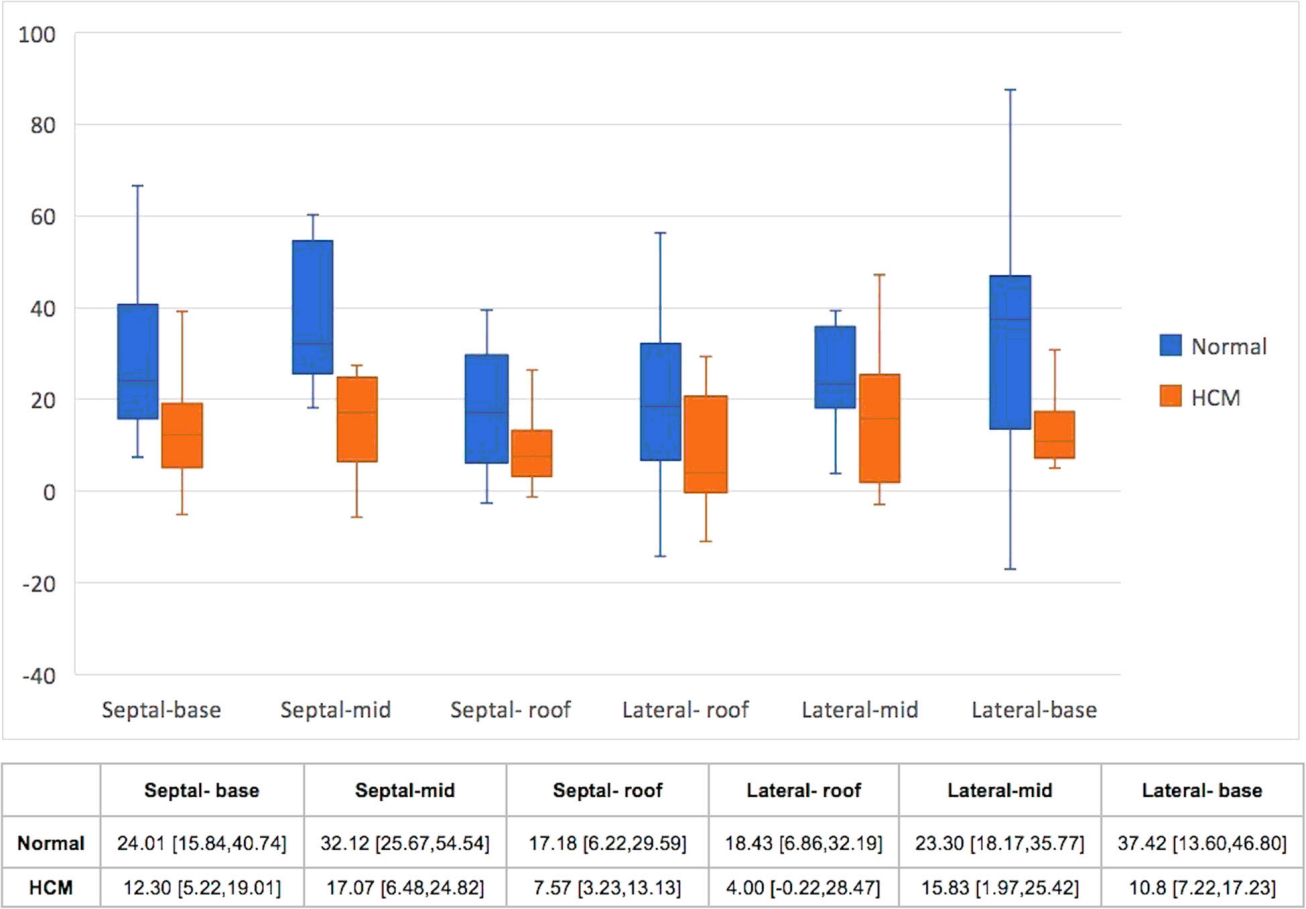
The Box and Whisker plot shows median and 25 to 75 percentile range of the atrial longitudinal strain in each region between the normal (blue) and hypertrophic cardiomyopathy (HCM) groups (orange)

The PALS was significantly lower in the HCM group than the control group (*P* < 0.001). The longitudinal strain of LA regions was significantly reduced in the HCM group compared to the normal group, except the septal-roof and the lateral- roof of the LA (Table 3) (Fig. 1).

**Table 3:** Two-dimensional echocardiographic data of cats in the control and hypertrophic cardiomyopathy groups

Variable	Control (*N* = 20)	HCM (*N* = 17)	*p*-value
**PALS**	27.25 [21.18,32.97]	13.33 [5.53,17.32]	< 0.001*
*Longitudinal strain of each left atrial region*			
Septal - base	24.01 [15.84,40.74]	12.30 [5.22,19.01]	0.006*
Septal - mid	32.12 [25.67,54.54]	17.07 [6.48,24.82]	< 0.001*
Septal - roof	17.18 [6.22,29.59]	7.57 [3.23,13.13]	0.063
Lateral - roof	18.43 [6.86,32.19]	4.00 [-0.22,28.47]	0.067
Lateral- mid	23.30 [18.17,35.77]	15.83 [1.97,25.42]	0.038*
Lateral- base	37.42 [13.60,46.80]	10.8 [7.22,17.23]	0.002*

*PALS* peak atrial longitudinal strain, *HCM* hypertrophic cardiomyopathy

Data are expressed as median and 25th, 75th percentiles

* indicate statistical significance at *p* < 0.05 by Mann-Whitney U Test

An assessment of the LA function of 3 subgroups [control group (*n* = 20), HCM cats with a LA diameter < 16 mm (*n* = 09), and HCM cats with a LA diameter ≥ 16 mm (*n* = 08)] was compared (Table 4). The results showed that LA-FS (*P* < 0.001), LA-EF (*P* = 0.001), FAC (*P* < 0.001) and PALS (*P* < 0.001) in both HCM cat subgroups were significantly lower than in the control group. However, the values of these variables were not significantly different between HCM cat subgroups.

**Table 4:** Assessment of left atrial function of 3 subgroups of cats

Variables	Control (*N* = 20)	HCM with LAD < 16 mm (*N* = 09)			HCM with LAD ≥ 16 mm (*N* = 08)	*P*-value	
LA-FS (%)	25.27 [23.29,31.67] ^a^		17.4 [8.68, 19.67] ^b^	14.26 [6.38, 20.00] ^b^			< 0.001*
LA-EF (%)	64.97 [59.75,83.05] ^a^		36.53 [28.07, 75.9] ^b^	39.87 [25.45, 60.41] ^b^			0.002*
FAC (%)	63.28 [57.46,72.31] ^a^		29.57 [14.66, 51.50] ^b^	32.09 [24.07, 52.39] ^b^			< 0.001*
PALS (%)	27.25 [21.18,32.97] ^a^		8.4 [5.53, 21.57] ^b^	14.50 [5.94, 18.34] ^b^			< 0.001*

*FAC* left atrial fractional area change, *HCM* hypertrophic cardiomyopathy, *LAD* left atrial diameter, *LA-EF* left atrial ejection fraction, *LA-FS* left atrial fractional shortening, *PALS* peak atrial longitudinal strain

Data are expressed as mean ± standard deviation

*indicate statistical significance at *P* < 0.05 of 3 subgroups

The significance difference was assessed by Kruskal Wallis test

^a^ and ^b^ indicate significant difference

The correlations between the peak atrial longitudinal strain and echocardiographic values assessed by conventional echocardiography in entire population showed weak positive correlations between PALS and peak velocity of early diastolic transmitral flow (r = 0.41, *P* = 0.012), peak velocity of systolic pulmonary vein flow (r = 0.41, *P* = 0.013), peak velocity of diastolic pulmonary vein flow (r = 0.341, *P* = 0.042), and peak velocity of early diastolic mitral annular motion as determined by pulsed-wave Doppler echocardiography (r = 0.459, *P* = 0.004). Moderate negative correlations between PALS and interventricular septum thickness at end-diastole (r=-0.563, *P* < 0.001) and left ventricular posterior wall thickness at end-diastole (r=-0.516, *P* = 0.001) were observed. There was no significant correlation between PALS and LAD (Table 5). There was also no correlation between PALS and heart rate (r=-0.042, *P* = 0.803).

**Table 5:** The correlation of the peak atrial longitudinal strain and echocardiographic values assessed by conventional echocardiography in entire population

Variable	r	*p*-value
**Size and structure**		
IVSd (mm)	-0.563	< 0.001*
LVIDd (mm)	0.362	0.028*
LVPWd (mm)	-0.516	0.001*
LA (mm)	-0.248	0.139
LA:Ao	-0.315	0.058
**LV function**		
FS%	-0.061	0.721
E (m/s)	0.41	0.012*
A (m/s)	0.277	0.107
E:A	-0.191	0.271
IVRT (m/s)	-0.204	0.240
S (cm/s)	0.41	0.013*
D (cm/s)	0.341	0.042*
AR (cm/s)	0.164	0.353
S:D ratio	0.164	0.338
E′ (cm/s)	0.459	0.004*
A′ (cm/s)	0.117	0.523
S′ (cm/s)	0.078	0.651
E′:A′ ratio	0.132	0.435
E:E′ ratio	-0.21	0.212

*Abbreviations*: *A *peak velocity of early diastolic transmitral flow, *A′ *peak velocity of diastolic mitral annular motion as determined by pulsed wave Doppler, *AR * Peak velocity of pulmonary vein flow reversal at atrial contraction, *D *Peak velocity of diastolic pulmonary vein flow, *E *Peak velocity of early diastolic transmitral flow, *E′ *Peak velocity of early diastolic mitral annular motion as determined by pulsed wave Doppler, *E:A *Ratio of E to A, *E′A′* Ratio of E′ to A′, *E:E′ *Ratio of E to E′, *FAC *Left atrial fractional area change, *FS *Left ventricular fractional shortening, *HCM  *Hypertrophic cardiomyopathy, *IVRT *Isovolumic (or isovolumetric) relaxation time, *IVSd *Interventricular septum thickness at end-diastole, *LA *Left atrium, *LA:Ao *Left atrial and aorta ratio, *LV  *Left ventricle, *LVIDd *Left ventricular internal dimension at end -diastole, *LVPWd *Left ventricular posterior wall thickness at end-diastole, *S *Peak velocity of systolic pulmonary vein, *S′* Peak velocity of systolic mitral annular motion as determined by pulsed wave Doppler, *S:D *Ratio of S to D

The significant correlation was assessed by Pearson’s correlation coefficient

*indicate statistical significance at *p* < 0.05

**Fig. 2:**
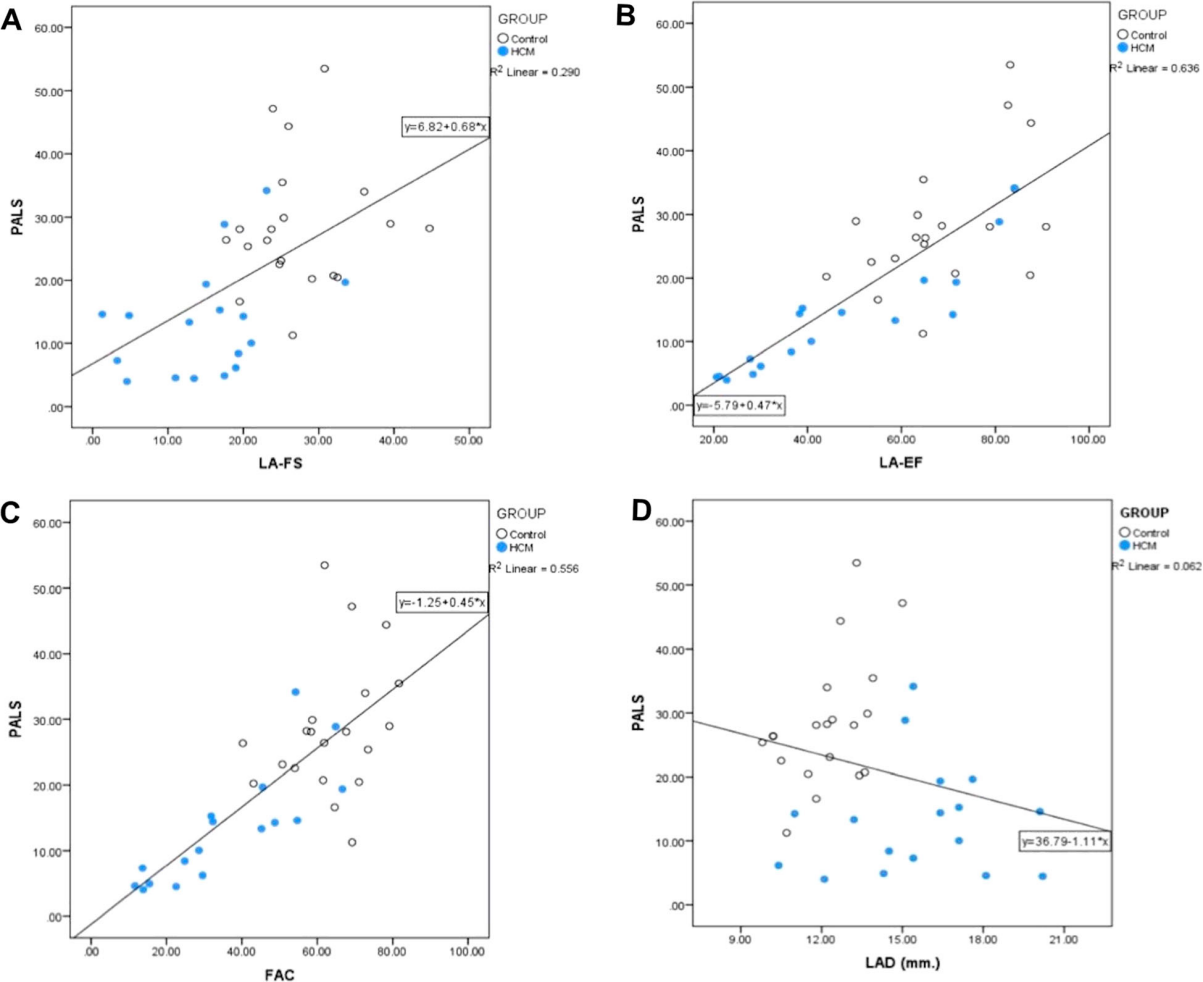
Correlations between PALS and other LA function parameters and LA diameter using Pearson’s correlation coefficient; **a**. PALS and LA-FS (r=0.538, *P*=0.001) **b.** PALS and LA-EF (r=0.797, P <0.001) **c.** PALS and FAC (r=0.746, *P* <0.001) **d. **PALS and LAD (r=-0.248, P =0.139) in the control (white) and hypertrophic cardiomyopathy groups (blue). PALS= peak atrial longitudinal strain; LA-FS= left atrial fractional shortening LA-EF= left atrial ejection fraction; FAC =left atrial fractional area change; LAD= left atrial diameter

The correlation analysis of data of all cats demonstrated a highly positive correlation between PALS and LA-EF (r = 0.797, *P* < 0.001) as well as PALS and FAC (r = 0.746, *P* < 0.001). The peak atrial longitudinal strain moderately correlated with LA-FS (0.538, *P* = 0.001) (Fig. 2).

The ANCOVA test demonstrated that age, sex and breeds had no effect on values of PALS, LA-FS and LA-EF. However, FAC was affected by breeds (domestic shorthair or pure breeds) (*P* = 0.04).

The intra-observer and inter-observer measurements of PALS variability were 4.17% and 14%, respectively.

## Discussion

We undertook this study to assess LA deformation of control cats and cats affected with HCM, using 2D-STE. The first major finding of this study was that 2D-STE is feasible for use in assessing LA deformation in cats. The second was that a difference in PALS was identified between cats with HCM and healthy cats. Finally, no correlation was found between PALS and LA size, but it correlated well with other echocardiographic parameters of LA function variables, including LA-FS, LA-EF and FAC.

Two-dimensional speckle tracking echocardiography is an echocardiographic technique that can be used to assess LA longitudinal strain in dogs [8, 9, 12, 31] and humans [10, 11, 27, 32]. Few studies using 2D-STE to assess left ventricular function in cats with HCM have been published [22, 23, 28–30]. This technique has been used to evaluate left ventricular myocardial function with adequate repeatability [22, 23, 28]. To our knowledge, no study focusing on assessment of LA function in feline HCM using 2D-STE has been reported. The present study demonstrated that 2D-STE may be used to evaluate changes in LA function in HCM cats with high repeatability and reproducibility. Intra- and inter-observer variability of PALS were clinically acceptable (CV < 15%), and similar results have been reported in humans [27] and dogs [8, 12].

The PALS, assessed by 2D-STE provides information on the longitudinal deformation of the LA during the reservoir phase [10, 11]. The result of this study showed that PALS was lower in the HCM group than the control group. In addition, LA-FS, LA-EF and FAC, conventional echocardiographic parameters for assessing LA contraction, were found to be lower in the HCM group than in the control group. This result is in agreement with previous studies reporting a reduction in LA function in cats affected with HCM, particularly those with congestive heart failure [5, 20, 33]. The present study showed that the LA roof had the lowest longitudinal strain. The LA roof is closed to the mediastinum, which may limit the movement of this region [26]. In humans, the strains of base and mid regions are similar [34]. Our study showed that the mid region had the highest strain. Further investigations are needed to clarify whether species variation is the cause of this difference. The longitudinal strains of all LA regions were decreased in the HCM group, suggesting global LA reservoir functional changes in cats affected with HCM. In this study, only one peak of atrial longitudinal strain was found in cats, which is different from that found in humans and dogs [8–11, 31]. This may occur secondary to rapid heart rate in cats. As in case of pulsed-wave Doppler echocardiography and tissue Doppler imaging, early and late diastolic transmitral flow and early and late diastolic mitral annular motion can be summated. Two cats from the control and HCM groups had early and late diastolic transmitral flow and mitral annular motion summation.

Interestingly, the present study showed that changes in PALS, LA-FS, LA-EF and FAC were found in HCM cats with both normal and enlarged LA size. A previous study showed a decrease in LA-EF in HCM cats with congestive heart failure with significant LA enlargement but not in asymptomatic HCM cats [5]. Whether a decrease in LA function is a consequence or cause of LA dilatation has to be proved. The results of our study suggest that the poor performance of the LA function in HCM cats may be not dependent on the LA size because the LA function and deformation have been detected in HCM cats both with and without LA enlargement.

The PALS assessed by 2D-STE correlated with LA function parameters assessed by conventional echocardiography. This result suggests that PALS offers an additional method for evaluating LA function. However, the load-dependency and high repeatability are limitations that must be addressed for the routine use 2D-STE [10, 11, 32, 34]. Based on the ANCOVA of data from this study, PALS was not affected by age, breed or sex. However, breed did affect FAC when assessed by conventional echocardiography. These findings suggest that PALS may be a more suitable technique for evaluating LA function in cats than some parameters assessed by conventional echocardiography. However, this study could not demonstrate the superiority of PALS as an early detection method because the decline in PALS was detected at the same time as the decreases in LA-FS, LA-EF and FAC. The usefulness of 2D-STE as an early detection method for the assessment of cardiac dysfunction needs further studies.

PALS also correlated with left ventricular wall thickness, chamber size and diastolic function parameters assessed by conventional echocardiography and tissue Doppler imaging. This finding suggests a relationship between left ventricular structural and functional changes and LA reservoir function.

The present study has some limitations that should be considered. First, the number of cats used was relatively small. Studies with a larger number of cats should be performed before adapting 2D-STE for use in clinical routine. Second, 2D-STE software was created for the analysis of left ventricular function in humans; therefore, it may have some limitations for LA function assessment in cats. In addition, the inter-vendor software variability is high; therefore, the results of this study can only be compared by using the same STE software. Third, during assessment it was challenging to trace the thin feline LA wall and therefore also some undesired tracking of extracardiac structure (such as mediastinum) could have occurred; however, increasing the size by increasing the depth and gain of images may help to visualize the LA wall. Fourth, myocardial wall dropout in the interatrial septum and pulmonary vein inlets may affect the tracking procedure. Tracing the LA cavity before atrial contraction may help to visualize these areas better [9]. Lastly, 2D-STE requires high-quality images that are difficult to obtain in cats with a very fast heart rate. The 2D-STE software used in the present study does not allow analysis in subjects with heart rate higher than 240 beats/minute. Therefore, cats had to be handled in low-stress conditions to help maintain a low heart rate. The use of an ultrasound machine system with higher frame rate may solve this problem [35]. Although a rapid heart rate may affect the image and tracking quality, the results of this study showed that PALS did not correlate with heart rate, suggesting that heart rate may not have a direct effect on PALS.

## Conclusions

Assessment of the PALS of the LA by 2D-STE is feasible to evaluate LA deformation in HCM cats. The method showed an acceptable repeatability and reproducibility. Studies with a larger number of HCM cats should be performed to confirm the advantages of 2D-STE in the assessment of LA function before the technique can be applied in the routine clinical settings.

## Methods

### Animals

The study population consisted of healthy cats and client-owned cats affected with HCM. All cats presented at Small Animal Teaching Hospital, Faculty of Veterinary Science, Chulalongkorn University, Thailand during August 2018 – June 2019. The study protocol was approved by the Animal Care and Use Committee, Faculty of Veterinary Science, Chulalongkorn University, Thailand (Animal Use Protocol No.1,831,073). The sample size for control and HCM groups (at least 17 cats per group) was calculated by the statistical software (G*Power test 3.1), using the estimated standard deviation of the percentage of the LA fractional shortening (LA-FS) from a previous study [5], an expected 80% power, and α value of 0.05, which was sufficient to determine a minimum difference in LA-FS between the two groups. LA-FS was chosen because of a lower standard deviation compared to other echocardiographic parameters.

### Cats

Adult cats (> 1 year old) with body weight 2–6 kilograms, any sex and breed were enrolled in the study. Clinical findings for all cats were recorded. All cats were subject to a complete physical examination, systolic blood pressure measurement and blood collection for complete blood count, blood chemistry and total T4 measurements. Cardiac examination including echocardiography, electrocardiography and thoracic radiography was performed without sedation in all cats. No cats had received medications before being enrolled in the study. Cats with renal disease (creatinine > 2.0 mg/dL), systemic hypertension (systolic blood pressure > 160 mmHg), hyperthyroidism (serum total T4 concentration > 4 µg/dl) [36] and any systemic diseases were excluded from the study.

Cats were divided into two groups: the control and HCM groups. Those in the control group consisted of healthy cats that had a left ventricular wall thickness of < 6 mm and an LA diameter of < 16 mm assessed by echocardiography and showed no other cardiovascular and systemic illness. Cats with left ventricular wall thickness of ≥ 6 mm during end diastole were recruited into the HCM group [4–7, 30, 36, 37]. Cats with and without congestive heart failure signs were accepted. For subgroups analysis, the population consisted of three subgroups (control group, HCM cats with a LA diameter < 16 mm, and HCM cats with a LA diameter ≥ 16 mm).

### Conventional echocardiography

Two-dimensional and M-mode echocardiography were performed by an investigator (SS). An ultrasound machine (Eko7, Samsung Medison, Seoul, South Korea) with a 4–12 MHz phased array transducer was used. M-mode echocardiography was performed on the right parasternal long-axis four-chamber view, to measure the chamber size and wall thickness by using a leading edge-to-leading edge technique. The M-mode cursor placed perpendicular to the interventricular septum and left ventricular wall below the tips of the mitral valves at the largest ventricular chamber size. Left ventricular internal dimension at end-diastole and end-systole, interventricular septum thickness at end-diastole and end-systole, left ventricular posterior wall thickness at end-diastole and end-systole were recorded. The percentage of left ventricular fractional shortening (FS%) was calculated by subtracting the left ventricular systolic dimension from the diastolic dimension dividing by the diastolic dimension. The ratio of LA to aorta dimension was measured during first diastolic frame of aortic valve closure from a right parasternal short axis view using two-dimensional echocardiography [20].

Pulsed-wave Doppler and tissue Doppler imaging were used for assessing left ventricular diastolic function [6, 22]. Transmitral flow velocities were measured from the left apical four-chamber view. The gate was placed at the tips of the mitral valve leaflets when they were wide open [38]. Peak velocity of early diastolic transmitral flow, peak velocity of late transmitral flow and the ratio of peak velocity of early diastolic to late diastolic transmitral flow was recorded. Isovolumic (or isovolumetric) relaxation time was measured from the left apical five-chamber view by placing the gate in the left ventricular outflow tract near the anterior mitral valve leaflet to reveal both aortic ejection flow and left ventricular inflow [37, 39]. Pulmonary vein flow velocities were measured in the right parasternal short-axis view [39, 40]. Peak velocity of systolic and diastolic pulmonary vein flow, and flow reversal at atrial contraction were recorded. The ratio of peak velocity of systolic to diastolic pulmonary vein flow were calculated. The myocardial motion along the longitudinal axis of the heart was investigated by placing the gate on the subendocardial portions of the lateral corner of the mitral annulus [24]. Peak velocity of early and late diastolic mitral annular motion and the ratio of peak velocity of early to late diastolic mitral annular motion as determined by pulsed-wave Doppler echocardiography were recorded. The ratio of peak velocity of early diastolic transmitral flow to peak velocity of early diastolic mitral annular motion were calculated [25]. The summation of early and late diastolic transmitral flows and early and late diastolic mitral annular motion assessed by pulsed-wave Doppler echocardiography was excluded from the analysis.

The LA diameter (LAD) was measured on the right parasternal long-axis four-chamber view parallel with the mitral annulus [40]. The maximal and minimal LAD (LADmax and LADmin) were measured. The LADmax was measured at end-systole (one frame before opening of mitral valve), and the LADmin was measured at peak atrial contraction (one frame before closure of mitral valve) [5]. Changes of the LAD were expressed as the percentage of fractional shortening of the left atrium (LA-FS) by the formula (LADmax-LADmin/LADmax) x100. The left apical four-chamber view was used to measure the maximal LA volume during atrial end-diastole and minimal LA volume during atrial end-systole. The percentage of LA ejection fraction (LA-EF) was calculated by an ultrasound machine automated software [5]. The LA area change (FAC) was measured by tracing the LA endocardial border during LA end diastolic and systolic phases on left apical four- chamber view. Left atrial maximal area (LAAmax) and minimal area (LAAmin) were measured in cm^2^. Then, FAC was then calculated with the formula FAC = [(LAAmax − LAAmin)/LAAmax] × 100 [8, 10]. The measurements of LA-EF and FAC were performed on the same cardiac cycle as 2D-STE.

### Two-dimensional speckle tracking echocardiography (2D-STE)

**Fig. 3:**
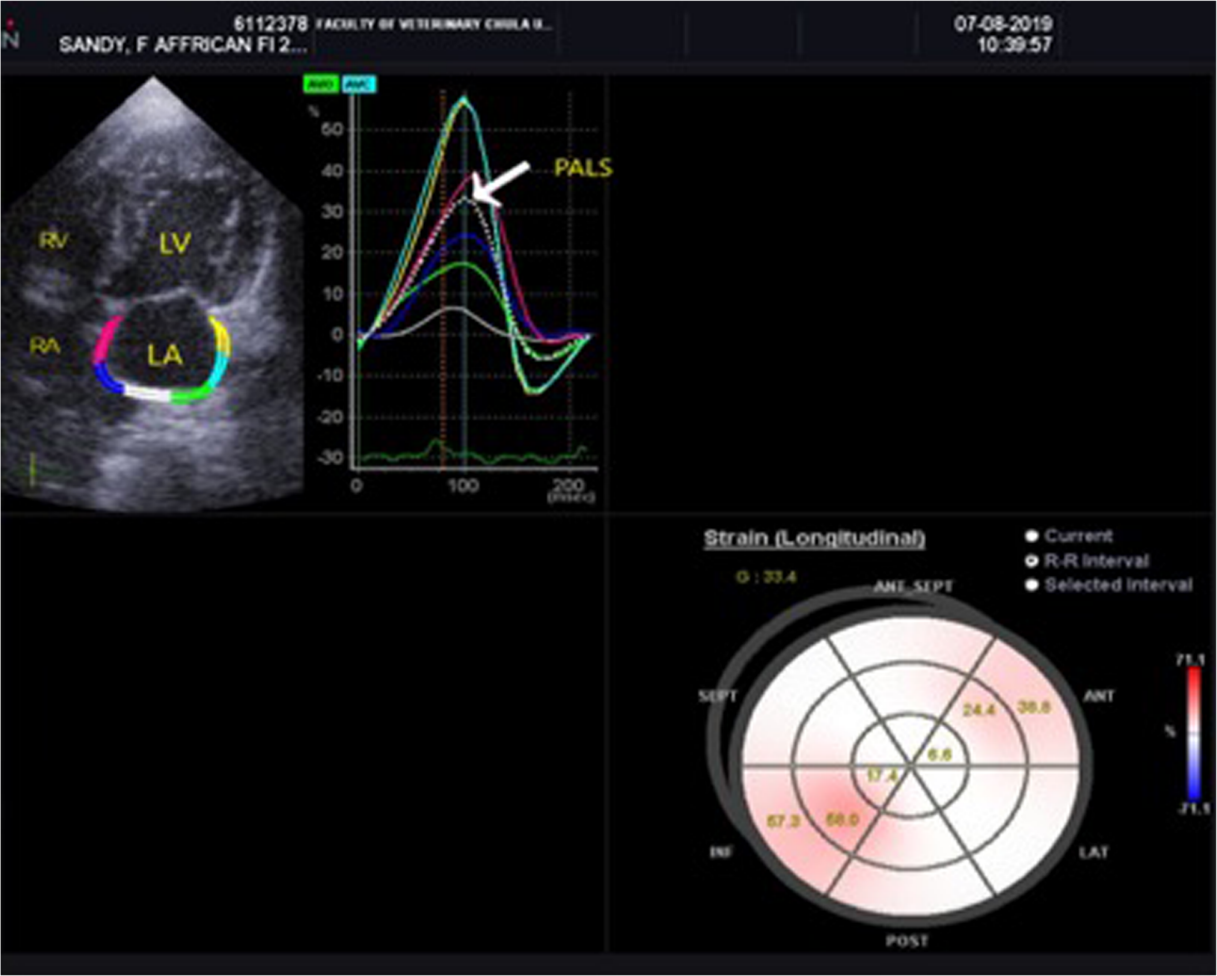
The image of the left apical four-chamber view of the left atrial strain profile of a hypertrophic cardiomyopathy cat. A region of interest is manually drawn to include the left atrial wall. The automatic software system divided the left atrial wall into 6 different segments with different colors. A white dotted line is presented as the mean of strain value of the left atrium. LA=left atrium; LV= left ventricle; PALS= peak atrial longitudinal strain (white arrow); RA= right atrium; RV= right ventricle

Two-dimensional speckle tracking echocardiography of the left apical four-chamber view was used to analyze the longitudinal deformation of the left atrium. Two-dimensional echocardiographic images were recorded at 100 frames/s for consecutive three cardiac cycles and three cineloops and stored these in the Digital Imaging and Communications in Medicine format. Offline analysis was performed in images with good quality from each cat. The LA wall, including the interatrial septum, the lateral wall and the atrial roof were tracked along during end-diastole. After automatic tracking, manual editing was performed to correct software errors in the region of interest. The ultrasound machine computer software (Strain 2.0 with Bull’s Eye) calculated the LA strain. The mean values of the measurements from three consecutive cardiac cycles were used in all analyses. Six segments were analyzed in each cat. The strain of each segment (as percentages) was plotted on the *y*-axis versus time (in seconds) on the *x*-axis over an entire cardiac cycle (Fig. 3). The different colored graphs represent strain curves from different segments. The white dotted line is the global strain. The QRS complex was used as the zero reference point, the peak positive longitudinal strain corresponds to the atrial reservoir function, the strain during early diastole represents atrial conduit function, and strain during late diastole corresponds to atrial contractile function. The peak atrial longitudinal strain (PALS) was the average of the peak of strain curves at the end of reservoir phase [8, 10]. The atrial strain rate was not analyzed in the present study.

### Measurement variability

The data from six randomly selected cats in the control group were used for calculating the variability of PALS. For the intra-observer variability, the measurement data from the same operator repeated on two different days (seven days apart) were used. Measurements were performed in the same cardiac cycle from the same cine loop. The inter-observer variability was calculated from measurements of two operators with different levels of experience in echocardiography (SS: Diplomate of the Asian College of Internal Medicine (Cardiology) and PhD, AK: MS). The variability was quantified as the coefficient of variation (CV) by the formula, %CV = standard deviation/mean x 100. The degree of repeatability was determined as follows: CV < 5%, very low variability; 5–15%, low variability; 16–25% moderate variability; or > 25% high variability [8, 41].

### Statistical analysis

Statistical analyses were performed using a commercially available software (SPSS version 22, Inc, Chicago, IL, USA). Descriptive statistics were used to describe the characteristics of the cats including sex, breed, age, body weight, systolic blood pressure and heart rate. The normality of data was assessed with the Shapiro-Wilks normality test. Comparisons between the two groups (the control and HCM cats) were performed by using the independent student t-test for normally distributed data and the Mann-Whitney U test for non-normally distributed data. The categorical data were compared by using Fisher’s exact test. Comparisons of 3 subgroups, including control and HCM cats with LAD < 16 mm, and the HCM cats with LAD > 16 mm, were performed by using the Kruskal-Wallis Test. The multiple comparisons were performed by the Duncan test method. An Analysis of Covariance model was used to test the fixed effects of sex, breed, and age, as covariates on conventional and 2D-STE-derived echocardiographic variables. The correlations between PALS and LA-FS, LA-EF, and FAC, assessed by conventional echocardiography, were tested by the Pearson’s correlation coefficient. A value of *P* < 0.05 was considered significant.

## Data Availability

The datasets used and/or analyzed during the current study are available from the corresponding author on reasonable request.

## Abbreviations

2D-STE: Two-dimensional speckletracking echocardiography
CV: Coefficient of variation
FAC: Fractional area change
HCM: Hypertrophic cardiomyopathy
LA: Left atrium (atrial)
LA-EF: The percentage of the LAejection fraction
LA-FS: The percentage of the LAfractional shortening
LAA: Left atrial area
LAD: Left atrial diameter
PALS: Peak atrial longitudinalstrain
